# TIMP-1: A Circulating Biomarker for Pulmonary Hypertension Diagnosis Among Chronic Obstructive Pulmonary Disease Patients

**DOI:** 10.3389/fmed.2021.774623

**Published:** 2022-02-25

**Authors:** Wenjun He, Chunli Liu, Jing Liao, Fei Liu, Hui Lei, Danmei Wei, Honglian Ruan, Bibhav Kunwar, Wenju Lu, Jian Wang, Tao Wang

**Affiliations:** ^1^State Key Laboratory of Respiratory Diseases, National Clinical Research Center for Respiratory Diseases, Guangzhou Institute of Respiratory Health, The First Affiliated Hospital of Guangzhou Medical University, Guangzhou, China; ^2^Guangdong Key Laboratory of Vascular Diseases, Guangzhou Medical University, Guangzhou, China; ^3^Department of Pulmonary Medicine, Amsterdam University Medical Center, Vrije Universiteit Amsterdam, Amsterdam, Netherlands; ^4^Department of Epidemiology and Biostatistics, School of Public Health, Tianjin Medical University, Tianjin, China; ^5^School of Public Health, Guangzhou Medical University, Guangzhou, China; ^6^Department of Clinical Medicine, Guangzhou Medical University, Guangzhou, China

**Keywords:** TIMP-1, pulmonary hypertension, chronic obstructive pulmonary disease, biomarker, diagnosis

## Abstract

Pulmonary hypertension (PH) is a common complication of chronic obstructive pulmonary disease (COPD) and induces increased mortality among COPD patients. However, there are no blood biomarkers to identify PH in COPD. Here, we investigated whether circulating angiogenic factors and cytokines could serve as (a) biomarker (s) for COPD-PH patients. Using Angiogenesis and Cytokine proteome profile array assay, we measured the level of 36 cytokines and 55 angiogenesis-associated proteins in plasma from four COPD patients with PH (COPD-PH) and four COPD patients without PH (COPD), respectively, tissue inhibitor of metalloproteinase 1 (TIMP-1) and thrombospondin 1(TSP-1) were significantly different between the two groups. Enzyme-linked immunosorbent assay (ELISA) was applied to measured TIMP-1 and TSP-1 in a validation cohort (COPD-PH, *n* = 28; COPD, *n* = 18), and TIMP-1 was the only factor that was significantly different between COPD-PH and COPD patients (*P* < 0.01). Logistic regression analysis demonstrated that elevated TIMP-1 was an independent risk factor for COPD-PH [odds ratio (OR) = 1.258, 95% CI: 1.005–1.574, *P* < 0.05). Next, we explored the expression level and function of TIMP-1 in human pulmonary arterial smooth muscle cells (hPASMCs) exposed to cigarette smoking extract (CSE, a major etiological factor of COPD). In cultured hPASMCs, CSE treatment increased both TIMP-1 protein level and cell proliferation, and exogenous TIMP-1 (25 ng/mL) treatment inhibited CSE-induced hPASMCs proliferation. Overall, our results indicated that TIMP-1 elevation could serve as a circulating biomarker to diagnose PH among COPD patients, and TIMP-1 elevation in COPD-PH could be adaptive.

## Introduction

Chronic obstructive pulmonary disease (COPD) is characterized by irreversible, progressive airflow obstruction. According to the World Health Organization, as of 2019, COPD was the third leading cause of death in both developing and developed countries (1–3). Pulmonary hypertension (PH) is a common complication of COPD and leads to cor pulmonale and even death (4, 5). The presence of PH in COPD (COPD-PH) patients leads to an increased risk of hospitalization, exacerbation (6), and mortality (7), and pulmonary arterial pressure (PAP) is negatively associated with life expectancy among COPD patients (8). Right heart catheterization (RHC), a gold standard of PH diagnosis, is invasive (9) and expensive (10), which makes it not feasible as a screening tool. Therefore, the development of screening tools is urgently demanding in the diagnosis of PH in COPD. Plasma biomarkers can be efficiently measured in an non-invasive manner, serving as an ideal screening tool for diseases. Deranged angiogenic signaling and inflammation contribute to the pathogenesis of PH (11–13), and altered circulating angiogenic factors and cytokines have been reported in pulmonary arterial hypertension patients (14–17). However, the changes in angiogenic factors or cytokines that are altered in PH among COPD patients are unknown. With an attempt to identify diagnostic blood biomarkers for PH in COPD, angiogenesis, and cytokines, proteome profile arrays were performed to screen the factors related to COPD-PH patients, and the identified biomarkers were further verified in a validation cohort.

## Materials and Methods

### Patients

All the COPD patients in this study were recruited based on the Global Initiative for COPD criteria. Patients with pulmonary arterial systolic pressure (PASP) ≥35 mmHg estimated by a designated Doppler Echocardiography (ECHO) were considered as COPD-PH. Among the COPD patients, 4 without PH (COPD group) and 4 with PH (COPD-PH group) were age- and gender-matched in the screening cohort; and 46 COPD patients were included in the validation cohort, 18 of them were without PH (COPD group), and 28 had PH (COPD-PH group). Subjects were recruited from the First Affiliated Hospital of Guangzhou Medical University. This study was approved by the Ethics Committee of the First Affiliated Hospital of Guangzhou Medical University (No. 2018-hs-120). All participants signed written informed consent forms before their inclusion in the study.

### Proteome Profiler Array Assay

Proteome Profiler assays were performed in the plasma from the screening cohort via Proteome Profiler Human Angiogenesis Array Kit (catalog #ARY007, R&D Systems, Inc., Minneapolis, MN, USA) and Proteome Profiler Human Cytokine Array Kit (catalog #ARY005B, R&D Systems) respectively according to the manufacturer's instructions. The array kits were designed to detect 55 angiogenesis-associated proteins and 36 cytokines in a single experiment by utilizing a sandwich immunoassay. Furthermore, pixel densities of the films were analyzed by Image J software (Image J software, 1.52a National Institutes of Health, Bethesda, MD).

### Enzyme-Linked Immunosorbent Assay (ELISA)

Concentrations of Tissue Inhibitor of Metalloproteinase 1 (TIMP-1) and Thrombospondin 1 (TSP-1) in the plasma were measured by Human TIMP-1 Quantikine ELISA Kit (catalog #DTM100, R&D Systems) and Human Thrombospondin-1 Quantikine ELISA Kit (catalog #DTSP10, R&D Systems) according to the manufacturer's instructions. All assays were performed in duplicate. The detection limits were 0.08 ng/mL for TIMP-1 and 0.944 ng**/**mL for TSP-1, respectively. Results were obtained by first measuring absorbance at 450 nm and then calculating cytokine concentration based on a standard curve.

### Preparation of Cigarette Smoking Extract (CSE)

According to a previously described protocol (18), CSE was freshly prepared from two Hongmei brand filtered cigarettes within 30 min before use. The acquired CSE suspension was yellowish with optical density (OD) 0.506 ± 0.008 at 405 nm. The CSE was adjusted to a pH value of 7.4 and filtered through a 0.22 μm filter, and the final product was considered a concentration of 100% for *in vitro* studies.

### Cell Culture

Human Pulmonary Artery Smooth Muscle Cells (hPASMCs) and smooth muscle cell medium (SMCM) were purchased from Sciencell (CA, USA; catalog #3110 and #1101). hPASMCs were placed in a CO_2_ incubator (5%, Thermo, USA) with a humidified atmosphere containing 5% CO_2_-95% air at 37°C in complete SMCM containing 5% fetal bovine serum (FBS, Gibco; Grand Island, NY, USA).

For CSE treatment, after cells had grown to 50–60% confluence, the culture media was replaced with SMCM basal culture media supplemented with 0.3% FBS for 12–24 h, and then exposed to 0, 0.125, 0.25, 0.5, 1, 2, 4% of CSE stimuli for 24, 36, 48 h, respectively.

### Cell Proliferation

Cell Counting Kit-8 assay (CCK-8, MCE company, New Jersey, USA) was used to detect cell proliferation following the manufacturer's recommendations. In brief, hPASMCs were seeded in 96-well plates at a density of 4×10^3^ cells per well and cultured 24 h in SMCM complete medium, then changed as SMCM containing 0.3% FBS for 12 h to allow cell arrest. After that, the cells were subjected to 0, 0.125, 0.25, 0.5, 1, 2, 4% of CSE for 24, 36, 48 h for cell proliferation assay.

### Western Blotting

Western blot was performed as previously described (19). Briefly, hPASMCs were washed in PBS and lysed in RIPA buffer supplemented with protease inhibitors on ice for 30 min. The supernatant was collected by centrifugation for 30 min with a speed of 12,000 rpm at 4°C. Protein concentrations were quantified by Bicinchoninic acid (BCA) Protein Assay Kit (catalog # 23227, Thermofisher Scientific) The protein samples were separated on 10% SDS-polyacrylamide gels and electrophoretically transferred onto PVDF membranes. Then the membrane was blocked in 3% non-fat milk in phosphate-buffered saline with Tween (PBST, 0.1% Tween 20) and incubated overnight at 4°C with anti-TIMP-1 primary antibody (1:500 dilution, catalog #A00561, Boster, Wuhan, China) and anti-β-actin antibody (1:2000 dilution, catalog #4695, Santa Cruz, CA, US), followed with secondary antibody incubation at room temperature for 2 h. Blots were developed using immobilon forte western horseradish peroxidase (HRP) substrate (WBLUF0100, Millipore) and then visualized using an enhanced chemiluminescence (ECL) reagent (KeyGen Biotechnology, Nanjing, China) and detected *via* ECL detection system (Tanon5200, ShangHai, China). The relative levels of immunoreactive proteins were quantified using the ImageJ software.

### Statistical Analysis

Continuous variables were presented as mean ± SEM. In studies comparing only two experimental groups, data were analyzed with two-sample independent Student's *t*-test to determine the significance. The logistic regression was used for univariate and multivariate analyses to test each independent factor for PH prediction. A multivariable logistic regression analysis, using the Forward: LR method (20), as performed using a final model to control potential confounding factors, and the statistical significance was set at the alpha = 0.05 level. OR and 95% CIs were calculated as the summary statistics. Besides, forest plots were generated to demonstrate OR and 95% CIs of the potential baseline prognostic covariates of COPD-PH. The level of statistical significance was taken as *P* < 0.05.

## Results

### Demographic Data and Clinical Characteristics of the Screening Cohort

For the eight COPD patients (4 with PH and 4 without PH, respectively) in the screening cohort, their baseline clinical characteristics were described in Table 1. All the COPD patients in the screening cohort were assessed by Doppler Echocardiography (ECHO), and those with PASP ≥ 35 mmHg were verified with Right Heart Catheterization (RHC, meam PAP ≥ 20 mmHg) for PH diagnosis. In the screening cohort, participants were divided into two groups based on PASP determined by ECHO: a group with PASP < 35 mm (*n* = 4, COPD group) and the other group with PASP ≥ 35 mm (*n* = 4, COPD-PH group). COPD-PH patients had a higher level of right atrium diameter (RAD) (COPD vs. COPD-PH, 28.00 ± 3.16 vs. 35.50 ± 2.64 mm, *P* < 0.05), and right ventricular diameter (RVD) (COPD vs. COPD-PH, 20.50 ± 1.19 vs. 24.50 ± 0.65 mm, *P* < 0.05) measured by ECHO than those without PH. There were no significant differences in age, sex, smoking, NT-pro-BNP, or predicted FEV_1_ (%) between the two groups (all *P* > 0.05).

**Table 1:** Clinical characteristics of participants for antibody array.

	**COPD**	**COPD with PH**	***P*** **-value**
*N*	4	4	-
Sex (male)	4 (100%)	4 (100%)	-
Age (year)	63.75 ± 3.84	63.50 ± 2.60	0.96
Smoking (pack-year)	23.75 ± 5.54	31.25 ± 1.25	0.24
NT-pro-BNP (pg/mL)	31.90 ± 23.83	121.6 ± 88.23	0.10
Predicted FEV_1_ (%)	37.60 ± 6.06	34.90 ± 4.70	0.74
EF (%)	72.50 ± 0.03	70.00 ± 0.03	0.57
RAD (mm)	28.00 ± 1.58	35.50 ± 1.32	0.01^*^
RVD (mm)	20.50 ± 1.19	24.50 ± 0.65	0.025^*^
PASP (mmHg)	29.50 ± 2.10	51.25 ± 4.11	<0.01^**^

*NT-pro-BNP, N-terminal pro-brain natriuretic peptide; predicted FEV_1_, predicted forced expiratory volume in 1 s; EF, ejection fraction; RVD, the right ventricular diameter measured by Doppler Echocardiography, RAD, the transverse diameter of right atrium measured by Doppler Echocardiography; PASP, systolic pulmonary arterial pressure measured by Doppler Echocardiography*.

* *P < 0.05 vs. COPD group,*

** *P < 0.01 vs. COPD group, absolute case numbers (% of cases in group). Values are shown as n (%) or mean ± SEM*.

**Figure 1:**
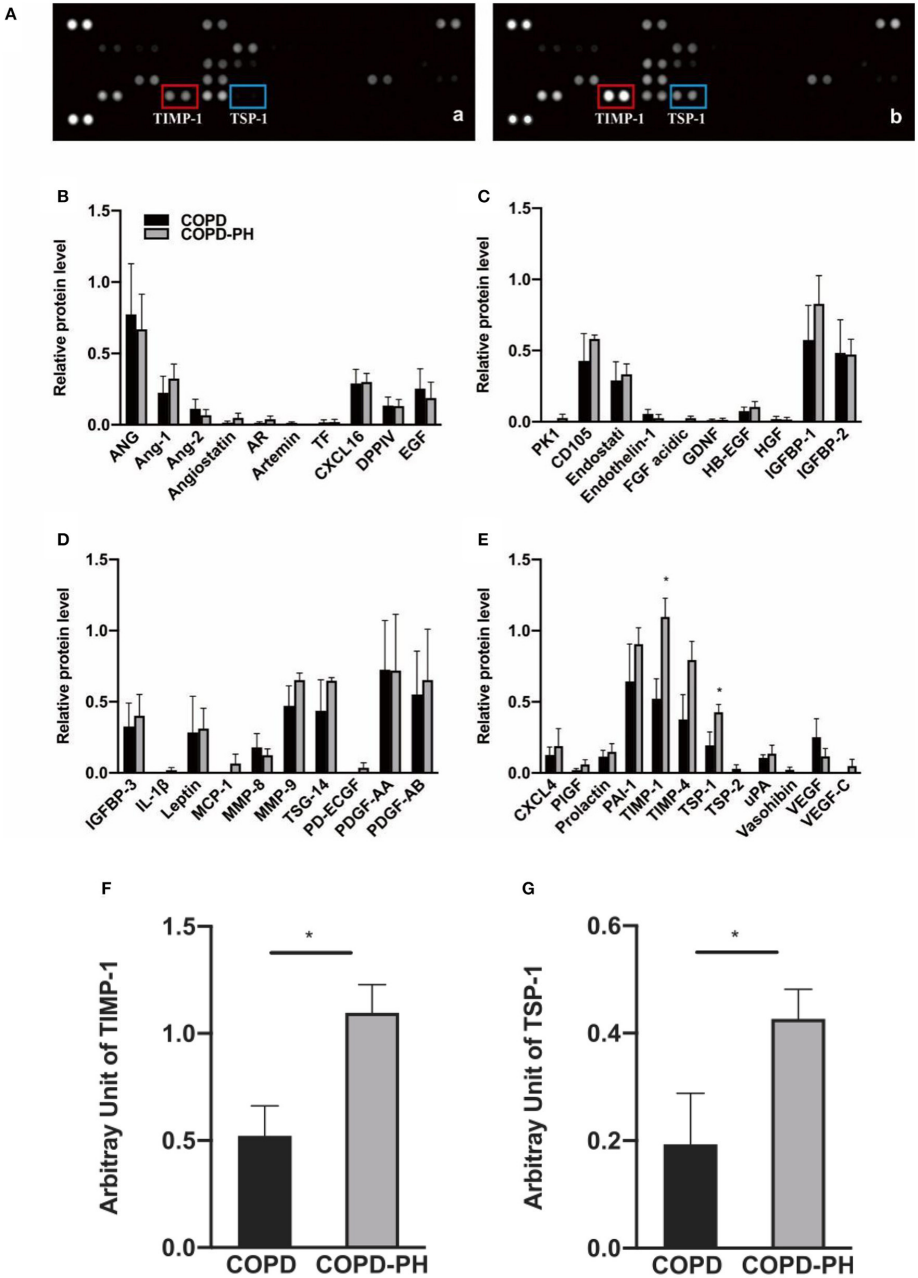
Angiogenesis array data. **(A)** Representative image of angiogenesis array of the plasma from COPD patients without PH [COPD, (a)] and with PH [COPD-PH, (b)] array blots. The levels of angiogenic factors are determined based on their blotting intensity in duplicates. **(B–E)** Relative protein level of for angiogenesis-associated protein determined by angiogenesis assay (Data were normalized to three pairs of reference spots). **(F,G)** Based on two-sample independent Student's *t*-test analysis, TIMP-1 and TSP-1 were significantly higher in COPD-PH than COPD group. Data were present as mean ± SEM (*n* = 4). ^*^P < 0.05 vs. COPD group.

We displayed two representative scanned images of COPD and COPD-PH patients in Figure 1A-a and Figure 1A-b, respectively. Using Proteome Profiler Human Angiogenesis Array Kit, as shown in Figure 1A, with a total of 55 angiogenesis-associated proteins, the following 42 proteins expressions were detected in the plasma of COPD and COPD-PH patients: ANG, Ang-1, Ang-2, Angiostatin, AR, Artemin, TF, CXCL16, DPPIV, EGF, PK1, CD105, Endostati, Endothelin-1, FGF acidic, GDNF, HB-EGF, HGF, IGFBP-1, IGFBP-2, IGFBP-3, IL-1β, Leptin, MCP-1, MMP-8, MMP-9, TSG-14, PD-ECGF, PDGF-AA, PDGF-AB, CXCL4, PlGF, Prolactin, PAI-1, TIMP-1, TIMP-4, TSP-1, TSP-2, uPA, Vasohibin, VEGF, and VEGF-C. Relative protein levels of these factors were showed in Figures 1B–E. Among these factors, the relative expression of Tissue Inhibitor of Metalloproteinases-1 (TIMP-1) (COPD vs. COPD-PH, 0.52 ± 0.28 vs. 1.1 ± 0.26, *P* < 0.05) Figure 1F, and Thrombospondin 1 (TSP-1) (COPD vs. COPD-PH, 0.19 ± 0.19 vs. 0.43 ± 0.11, *P* < 0.05) Figure 1G, were significantly higher in COPD-PH patients. The rest of the factors did not show a significant difference between the two groups.

For cytokine profiles, as shown in Supplementary Figure 2, with a total of 36 cytokines, the following 15 proteins expressions were detected in the plasma of COPD and COPD-PH patients: CCL5/RANTES, CD40 Ligand/TNFSF5, Complement Component C5/C5a, CXCL1/GROα, CXCL10/IP-10, CXCL11/I- TAC, CXCL12/SDF-1, G-CSF, ICAM-1/CD54, IL-1ra/IL-1F3, IL-13, IL-16, IL-18/IL-1F4, MIF, and Serpin E1/PAI-1. However, the difference did not reach statistical significance in the level among any of these cytokines between the two groups (*P* > 0.05).

### Demographic Data and ELISA Results of the Validation Cohort

As shown in Table 2, there was no statistical significance in sex, age, and smoking status between the two groups (COPD, *n* = 18; COPD-PH, *n* = 28) in the validation cohort. Results of ECHO showed lower ejection fraction (EF) values (COPD vs. COPD-PH, 71.20 ± 4.824 (%) vs. 66.32 ± 8.47 (%), *P* < 0.05), higher PASP (COPD vs. COPD-PH, 30.39 ± 3.65 vs. 49.86 ± 11.97 mmHg, *P* < 0.01), RAD (COPD vs. COPD-PH, 31.06 ± 3.78 vs. 36.86 ± 7.276 mm) and RVD (COPD vs. COPD-PH, 20.83 ± 2.09 vs. 24.54 ± 6.13 mm) in COPD-PH group when compared to COPD group. Besides, there was no statistical significance in predicted FEV_1_ value or NT-pro-BNP between the two groups.

**Table 2:** Clinical characteristics of patients for ELISA validation.

	**Patients with COPD**	**Patients with COPD-PH**	***P*** **-value**
	(n, M, M% = 18, 18, 100%)	(n, M, M% = 28, 25, 89.28%)	
Age (year)	66.28 ± 5.81	66.25 ± 6.75	0.49
Smoking (pack-year)	28.17 ± 3.53	25.54 ± 3.01	0.58
NT-pro-BNP (pg/mL)	114.9 ± 148.50	478.30 ± 1,265	0.12
FEV_1_/FVC (%)	47.69 ± 2.748	44.72 ± 2.12	0.39
Predicted FEV_1_ (%)	41.14 ± 11.03	36.49 ± 9.98	0.15
EF (%)	71.28 ± 4.82	66.32 ± 8.47	<0.05^*^
RAD (mm)	31.06 ± 3.78	36.86 ± 7.276	<0.01^**^
RVD (mm)	20.83 ± 2.09	24.54 ± 6.13	<0.01^**^
PASP (mmHg)	30.39 ± 3.65	49.86 ± 11.97	<0.01^**^

*M, the number of Male; M%, the percentage of Male; NT-pro-BNP, N-terminal pro-brain natriuretic peptide; FEV_1_/ FVC, Tiffeneau index; predicted FEV_1_, predicted forced expiratory volume in 1 s; EF, ejection fraction; RVD, right ventricular diameter measured by Doppler Echocardiography, RAD, the transverse diameter of right atrium measured by Doppler Echocardiography; PASP, systolic pulmonary arterial pressure measured by Doppler Echocardiography*.

* *P < 0.05 vs. COPD group,*

** *P < 0.01 vs. COPD group, absolute case numbers (% of cases in group). Values are shown as n (%) or mean ± SEM*.

**Figure 2:**
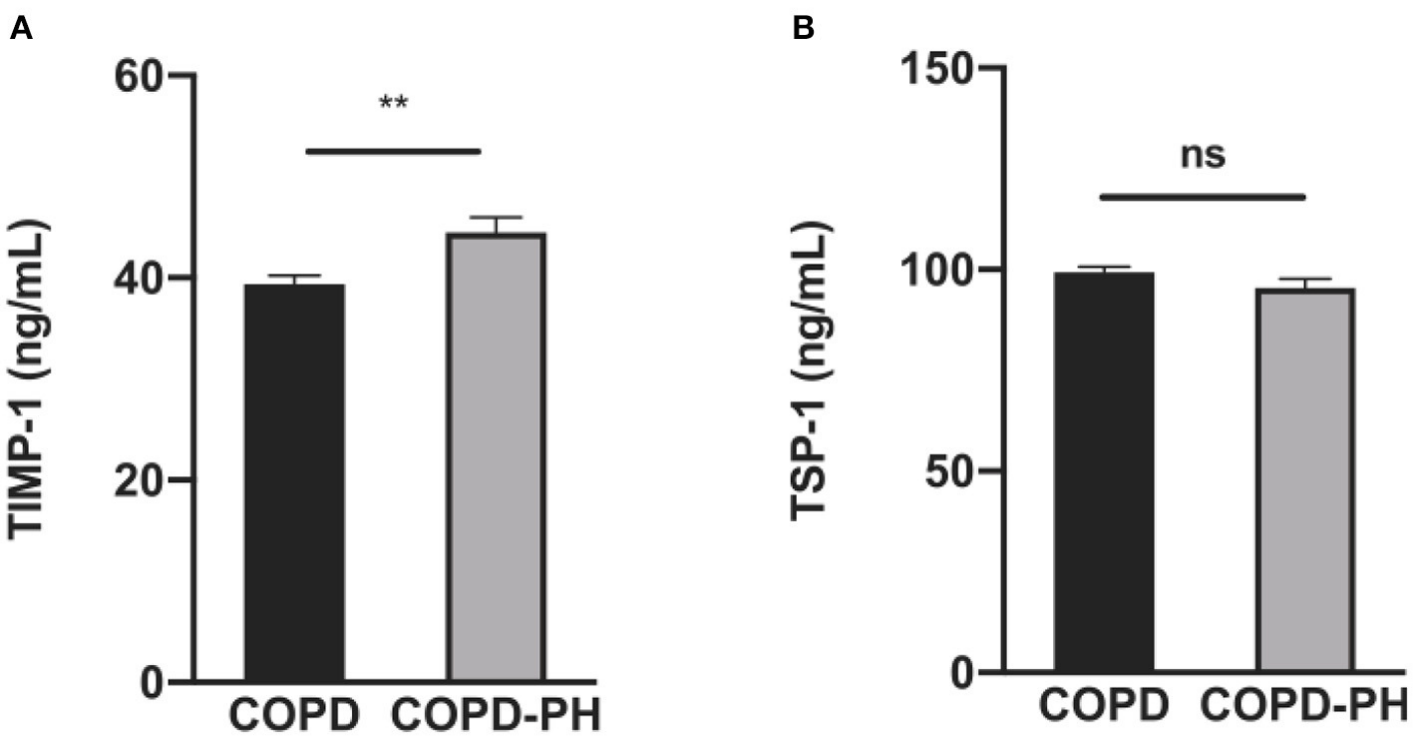
The results of TIMP-1 **(A)** and TSP-1 **(B)** between COPD and COPD-PH group in validation cohort by ELISA. The *P*-value of each factor was obtained from unpaired Student's *t*-test analysis. **P < 0.01 vs. COPD group; ns, no significant statistical difference.

Results from ELISA of the plasma indicated that in the validation cohort, TIMP-1 level in COPD-PH patients was significantly higher (COPD vs. COPD-PH, 39.40 ± 3.493 vs. 44.48 ± 7.795 ng/mL, *P* < 0.01, Figure 2A). However, the plasma level of TSP-1 is not statistically different between the two groups (COPD vs. COPD-PH, 99.34 ± 5.448 vs. 95.38 ± 12.01 ng/mL, *P* > 0.05, Figure 2B).

### Plasma TIMP-1 Is a Biomarker of PH Among COPD Patients

**Figure 3:**
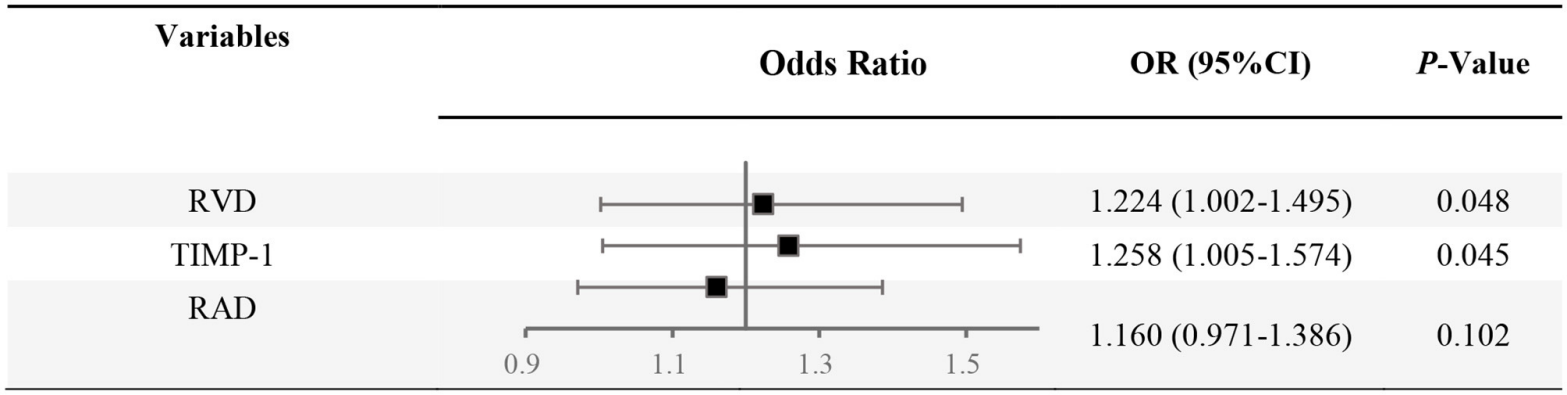
Forest plot of combined analysis on PH identification among COPD. OR, odds ratio; RVD, the right ventricular diameter measured by Doppler Echocardiography; RAD, the transverse diameter of right atrium measured by Doppler Echocardiography.

Multivariable analysis based on Logistic Regression Analysis was used to construct a predictive model considering the interaction between various confounding factors and estimate the probability of PH among COPD patients (Table 3 and Figure 3). The multivariable analysis considered age, smoking, sex, NT-pro-BNP level, RAD, and RVD as confounding factors in the verification cohort. Following adjustment for confounding factors, Logistic Regression Analysis demonstrated the elevation of TIMP-1 (OR, 1.258; 95% CI, 1.005–1.574; *P* < 0.05) and RVD (OR, 1.224; 95% CI, 1.002–1.495; *P* < 0.05) as independent predictive factors for the diagnosis of PH among COPD patients.

**Table 3:** Factors for PH diagnosis among COPD patients based on logistic regression analysis.

	**β**	**S.E**.	**Wald**	**df**	***P***	**OR**	**95% CI. for OR**
RVD	0.202	0.102	3.901	1	0.048	1.224	1.002–1.495
TIMP-1	0.229	0.114	4.022	1	0.045	1.258	1.005–1.574
RAD	0.148	0.091	2.668	1	0.102	1.160	0.971–1.386
Constant	−18.405	6.137	8.995	1	0.003	0.000	

*The P-value is the test of significance of the Wald statistic (calculated as the estimated coefficient (β) ratio to SEM^2^). df, Degrees of freedom; OR, odds ratio; 95% CI, 95% Confidence Interval*.

### CSE Increases TIMP-1 and Cell Proliferation, and TIMP-1 Reduces CSE-Induced Cell Proliferation in hPASMCs

**Figure 4:**
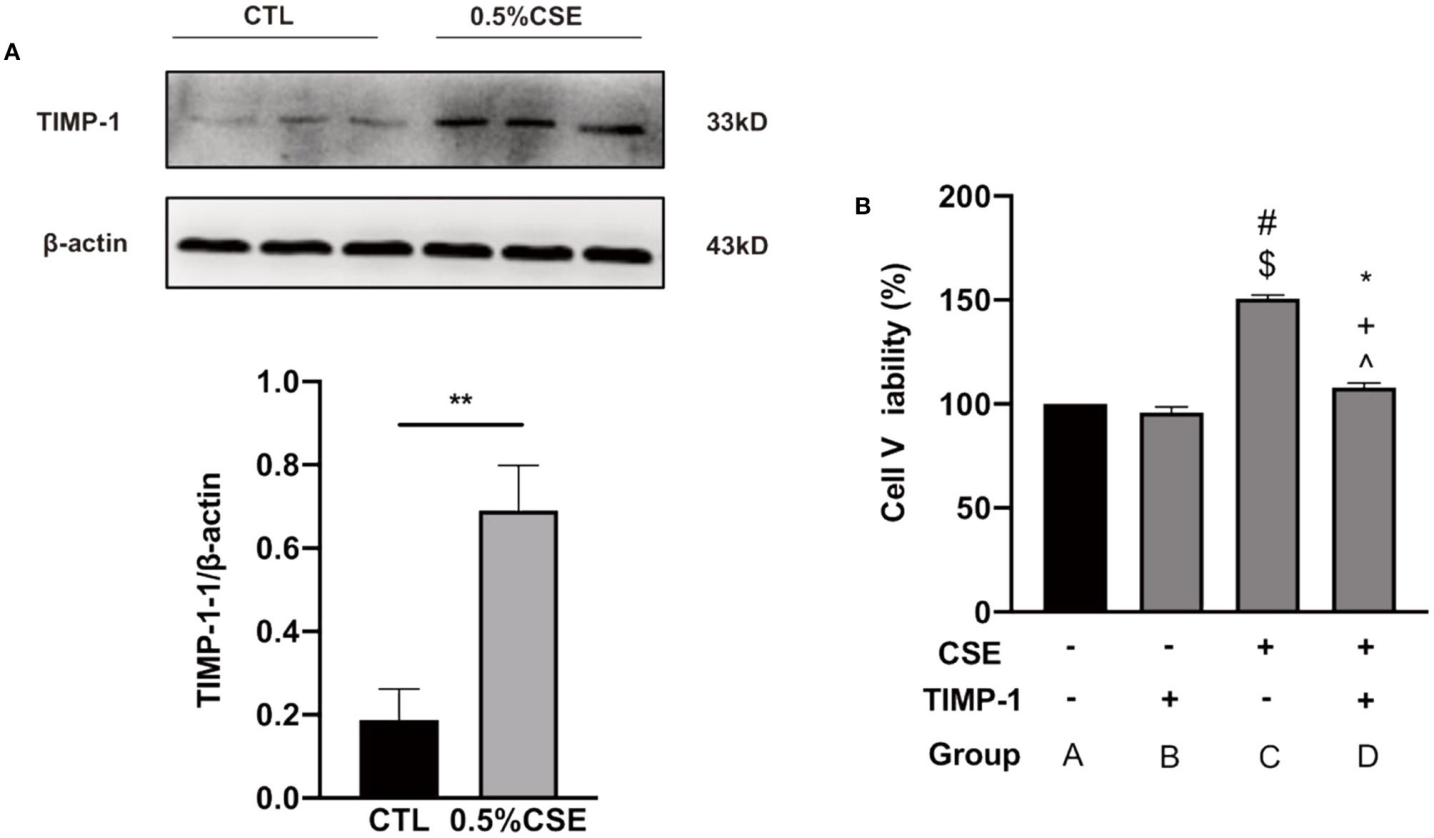
CSE increased TIMP-1 level in cultured hPASMCs, and TIMP-1 decreased hPASMCs proliferation under CSE. **(A)** Western Blotting indicates 0.5% CSE treatment for 36 h increased TIMP-1 expression in hPASMCs. **(B)** TIMP-1 attenuated CSE-stimulated hPASMCs proliferation. CSE, Cigarette Smoking Extract; hPASMCs, human Pulmonary Arterial Smooth Muscle Cell, **P < 0.01 vs. Control group. TIMP-1, tissue inhibitor of metalloproteinase-1, CSE “+,” with the treatment of CSE; CSE “−,” without treatment of CSE; TIMP-1 “+,” with the treatment of TIMP-1; TIMP-1 “−,” without treatment of TIMP-1; ^**#**^*P* < 0.01 vs. GroupA, ^$^*P* < 0.01 vs. Group B, *P < 0.05 vs. Group A, ^**+**^*P* < 0.05 vs. Group B, ^**∧**^*P* < 0.01 vs. Group C, *N* = 6.

As shown in Supplementary Figure 3, hPASMCs proliferation peaked at 36 h under 0.5% cigarette smoke exposure (CSE) treatment among a series of CSE concentrations and time points. Under this condition of CSE treatment, TIMP-1 expression in hPASMCs was significantly upregulated as determined by western blot (*P* < 0.01, Figure 4A), and hPASMCs proliferation was also increased significantly (*P* < 0.01, Figure 4B). In addition, under CSE exposure, TIMP-1 (25 ng/mL) treatment reduced hPASMCs proliferation significantly (*P* < 0.01, Figure 4B).

## Discussion

**Figure 5:**
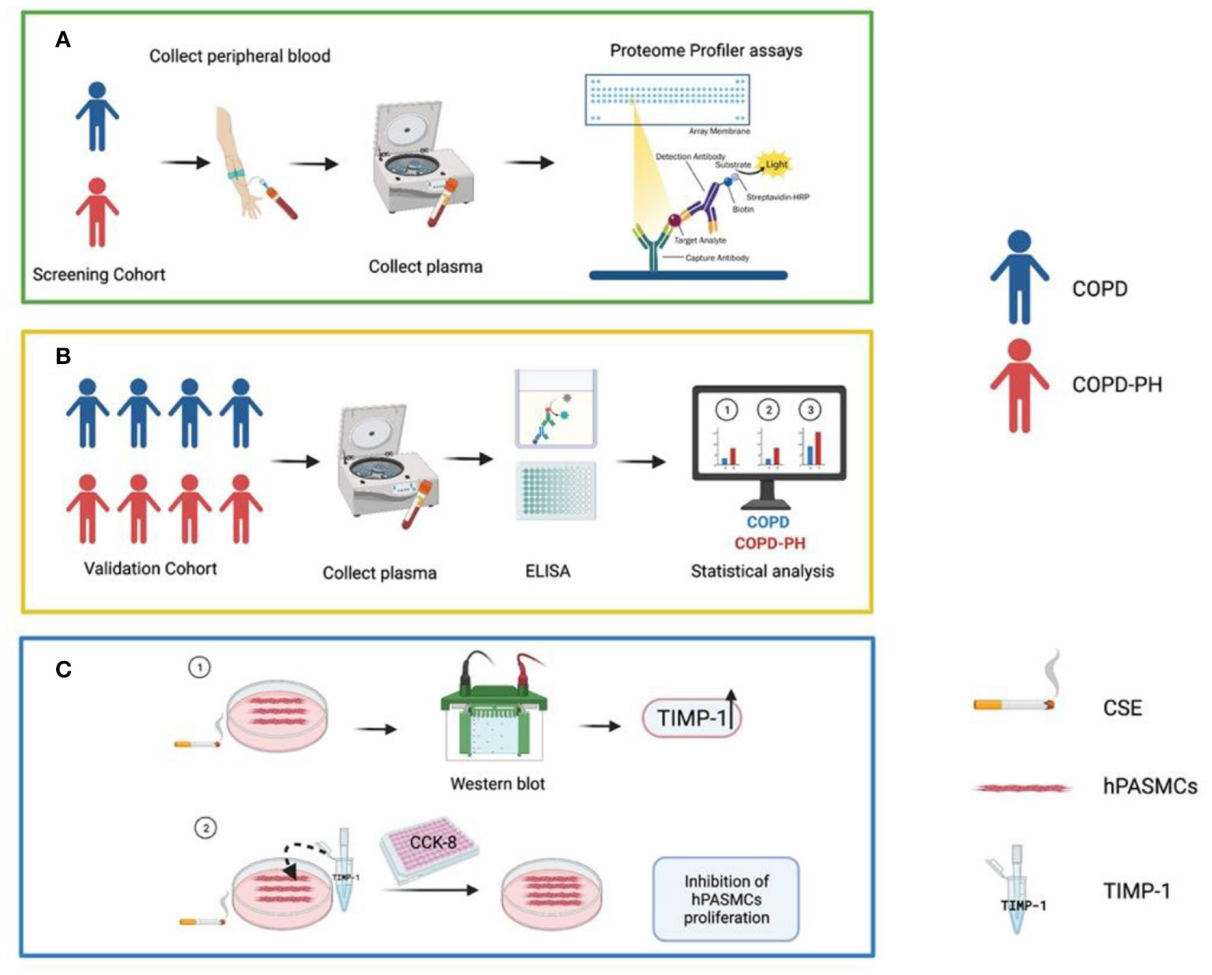
Summary figure of the schematic workflow of the current study. **(A)** Initial screening of angiogenic factors and cytokines. **(B)** ELISA verification in validation cohort and related statistical analysis. **(C)** Involvement of TIMP-1 in the hPASMCs mechanism. COPD, Chronic obstructive pulmonary disease; COPD-PH, Chronic obstructive pulmonary disease associated pulmonary hypertension; CSE, Cigarette smoke exposure; ELISA, Enzyme-Linked Immunosorbent Assay; hPASMCs, Human Pulmonary Artery Smooth Muscle Cells; TIMP-1, Tissue Inhibitor of Metalloproteinases-1. This figure was created using BioRender.

PH is a common complication of COPD, especially in those with severe lung function impairment (4, 8), which may eventually progress to cor pulmonale and even death (21). However, diagnosis of PH among COPD patients is technically challenging. Screening from a total of 55 angiogenesis-associated proteins and 36 cytokines, our study indicated that plasma levels of TIMP-1 could serve as a diagnostic index for PH among COPD. The schematic workflow of the current study is shown in Figure 5.

TIMP-1 is a crucial regulator of extracellular matrix degradation (22), and circulating TIMPs have been shown to be altered in several cardiovascular diseases (23). An early study shows that increased TIMP-1 levels is a risk factor of mortality among congestive heart failure patients (24). Recently, it has been demonstrated that PH patients with TIMP-1 elevation have an increased risk for death (25). In COPD patients, TIMP-1 levels are also increased (26, 27), and plasma TIMP-1 concentration is associated with the severity of COPD (27). Although elevated TIMP-1 concentrations have been demonstrated in PH or COPD in several previous studies, the changes of TIMP-1 in COPD patients with PH remain unknown. Here, we indicated that elevated TIMP-1 could be used to diagnose PH among COPD patients for the first time.

Cigarette smoke (CS) exposure is the most common etiological factor of COPD (4, 28–30) and also a risk factor for pulmonary vascular damages, which may lead to PH (31). In consistent, our study demonstrated that CSE treatment increased PASMCs proliferation, a characteristic pathological change of PH, and CSE also increased TIMP-1 in cultured PASMCs. TIMP-1 reduces pulmonary arterial pressure and attenuates pathological pulmonary vascular remodeling in monocrotaline-induced PH rat models. However, the mechanisms of PH development in COPD are different and could be more complicated. Additionally, the COPD-PH animal model has not been reported in previous studies to the best of our knowledge. Therefore, we tested the effects of TIMP-1 in cultured PASMs under CSE and found that TIMP-1 inhibits CSE-induced PASMCs proliferation, which may suggest that TIMP-1 elevation could be protective in COPD-PH patients.

Matrix metalloproteinases (MMPs) are zinc-dependent endopeptidases that play crucial roles in embryonic development, angiogenesis, arthritis, cardiovascular diseases, and cancer (32). MMPs are also involved in angiogenesis by mediating tissue remodeling and penetration of the extracellular matrix (33). The activity of MMPs is mainly inhibited by the presence of endogenous Tissue Inhibitor of Metalloproteinase (TIMPs) (34). Lepetit H's study showed that MMP-TIMPs imbalance (increased TIMP-1 and decreased MMP-3) contributed to smooth muscle cell proliferation in idiopathic pulmonary arterial hypertension (35). Among TIMPs, TIMP-1 preferentially inhibits MMP-9 (36). Zhu et al. demonstrated that MMP-2 and MMP-9 aggravate pulmonary arterial hypertension by increasing migration and proliferation in pulmonary endothelial cells (37), suggesting TIMP-1 may exert protective effects in PH. However, in our current study, elevated TIMP-1 was found in the plasma of COPD-PH patients compared to COPD patients without PH. However, there was no statistically significant difference in MMP-9 between the two groups, suggesting TIMP-1 is simply the biomarker of COPD-PH and did not exert protective effects *via* reducing MMP-9 in COPD-PH.

In another study, Zhang et al. screened 440 cytokines using the Human Cytokine Antibody Array among 20 COPD and 20 COPD-PH patients and found that MCP-4, CCL28, and CD40 were altered in the serum of COPD patients with PH (38). Together with our findings, it may also suggest multiple factors are involved in PH development in COPD patients. The mechanisms of PH development in COPD could be complicated; future studies are needed for in-depth knowledge of PH diagnosis and treatment among COPD patients.

Several limitations of our study merit description. Firstly, this is a single-center study. To maintain the freshness of blood, we attempted to choose the peripheral blood samples that were collected recently. Although the number of patients is limited, we found that TIMP-1 is a circulating marker of PH in COPD patients. Moreover, as there is no present COPD-PH animal model, we only investigated the role of TIMP-1 in the context of COPD *in vitro* through CSE- stimulated hPASMCs. Therefore, we believe it is fair to assume that the reported differences in the expression and/or activity of TIMP-1 were rather related to the manifestation of COPD-PH.

## Conclusion

Plasma TIMP-1 levels may serve as a non-invasive index to diagnose PH among COPD patients; TIMP-1 inhibits CSE-induced hPASMCs proliferation, suggesting TIMP-1 elevation in COPD-PH patients could be adaptive.

## Data Availability Statement

The original contributions presented in the study are included in the article/Supplementary Material, further inquiries can be directed to the corresponding author/s.

## Ethics Statement

The studies involving human participants were reviewed and approved by the Ethics Committee of the First Affiliated Hospital of Guangzhou Medical University (2018-hs-120). Informed consent was obtained from all subjects involved in the study. In addition, written informed consent has been obtained from the patients to publish this paper.

## Author Contributions

WH, TW, and JW conceived and designed the study. CL provided study materials and patients. JL, FL, HL, WL, and BK analyzed and interpreted the data and wrote the paper. DW and HR provided their statistical assistance. All authors have read and agreed to the published version of the manuscript.

## Funding

This work was supported by grants from the National Key Research and Development Program of China (2016YFC1304102), the National Natural Science Foundation of China (81700426, 81970057, 81630004, and 81970046), Guangdong Outstanding Young Scientist Funding (2021B1515020006), Zhongnanshan Medical Foundation of Guangdong Province (ZNSA-2020013 and ZNSA-202003), State Key Laboratory of Respiratory Disease (SKLRD-MS-201901), and the Science and Technology Program of Guangzhou, China (201904010329).

## Conflict of Interest

The authors declare that the research was conducted in the absence of any commercial or financial relationships that could be construed as a potential conflict of interest.

## Publisher's Note

All claims expressed in this article are solely those of the authors and do not necessarily represent those of their affiliated organizations, or those of the publisher, the editors and the reviewers. Any product that may be evaluated in this article, or claim that may be made by its manufacturer, is not guaranteed or endorsed by the publisher.

## Abbreviations

CCK-8: Cell Counting Kit-8 assay
CI: Cardiac Index
CO: Cardiac Output
COPD: Chronic obstructive pulmonary disease
COPD-PH: Chronic obstructive pulmonary disease associated pulmonary hypertension
CS: Cigarette Smoke
CSE: Cigarette smoke exposure
df: Degrees of freedom
ECHO: Doppler Echocardiography
EF: ejection fraction
FBS: fetal bovine serum
FEV1/FVC%: Forced expiratory volume in 1 s (FEV1)/Forced volume vital capacity (FVC) ratio
predicted FEV1: predicted forced expiratory volume in 1 s
hPASMCs: Human Pulmonary Artery Smooth Muscle Cells
mPAP: mean Pulmonary Arterial Pressure
PASP: Pulmonary Arterial Systolic Pressure
NT-pro-BNP: N-terminal pro-brain natriuretic peptide
OR: odds ratio
PH: Pulmonary hypertension
RAD: the transverse diameter of right atrium measured by Doppler Echocardiography
RHC: Right Heart Catheterization
RVD: the diameter of right ventricular measured by Doppler Echocardiography
SaO_2_: Oxygen saturation in the arterial blood
SMCM: smooth muscle cell medium
TIMP-1: Tissue Inhibitor of Metalloproteinases-1
TSP-1: Thrombospondin 1
95% CI: 95% Confidence Interval.
