# Supplementary material for: TIMP-1: A Circulating Biomarker for Pulmonary Hypertension Diagnosis Among Chronic Obstructive Pulmonary Disease Patients
Source: Front Med (Lausanne). 2022 Feb 25;8:774623. doi: 10.3389/fmed.2021.774623 (PMC8914225; doi:10.3389/fmed.2021.774623)
Supplement: Supplementary file 1 [file Data_Sheet_1.docx]

**Supplementary Materials**


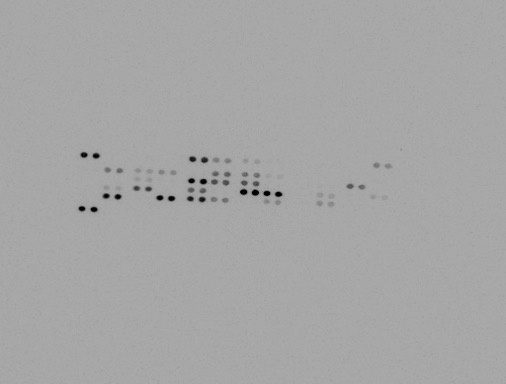

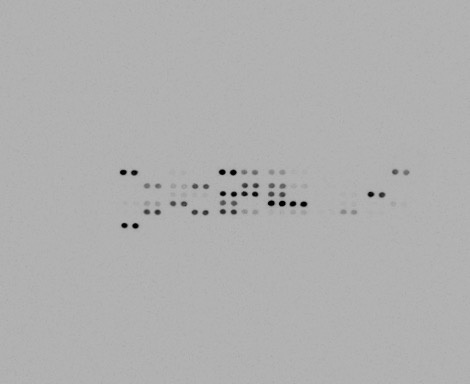

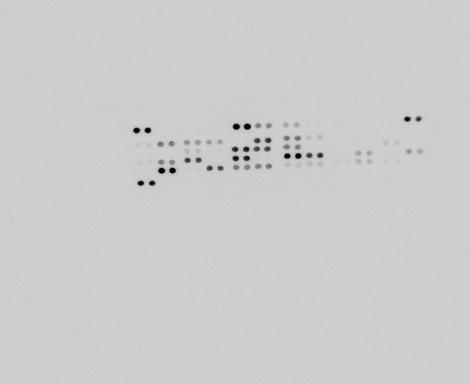

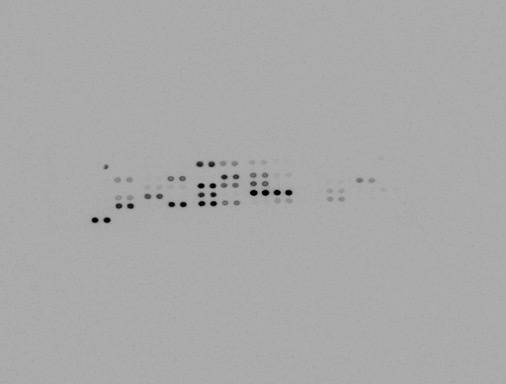

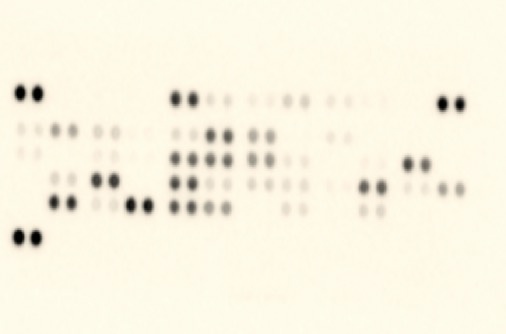

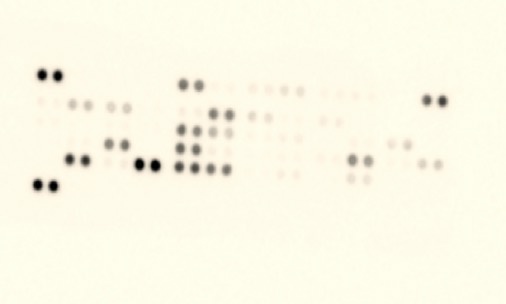

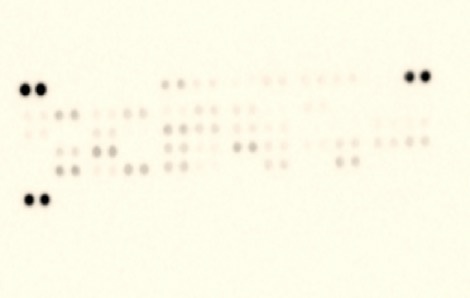

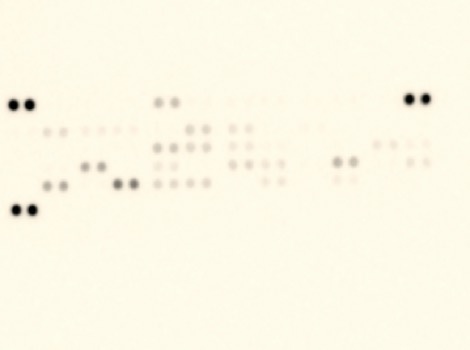


COPD without PH

COPD-PH

**Figure S1** The whole COPD-PH plasma Angiogenesis profiles. In the profiles of antibody arrays, the levels of Angiogenesis factors are proportional to their blotting intensity.

**
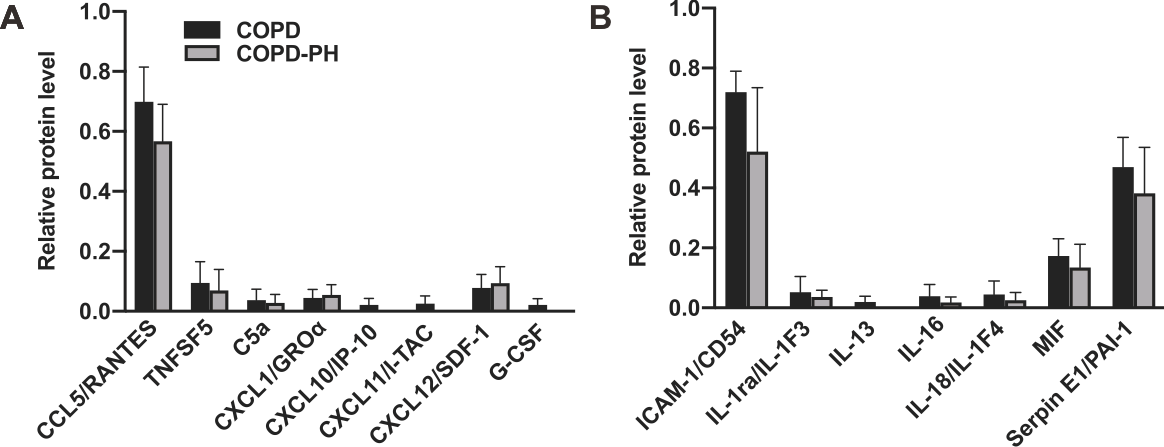
**

**Figure S2** Cytokine Array data**.** Based on two-sample independent Student’s t-test analysis results, no protein was identified and shown by a bar chart. The centerline in the bar chart indicates the standard deviation in each group. *P*$>$0.05 *vs.* Control group, N $=$4.

**
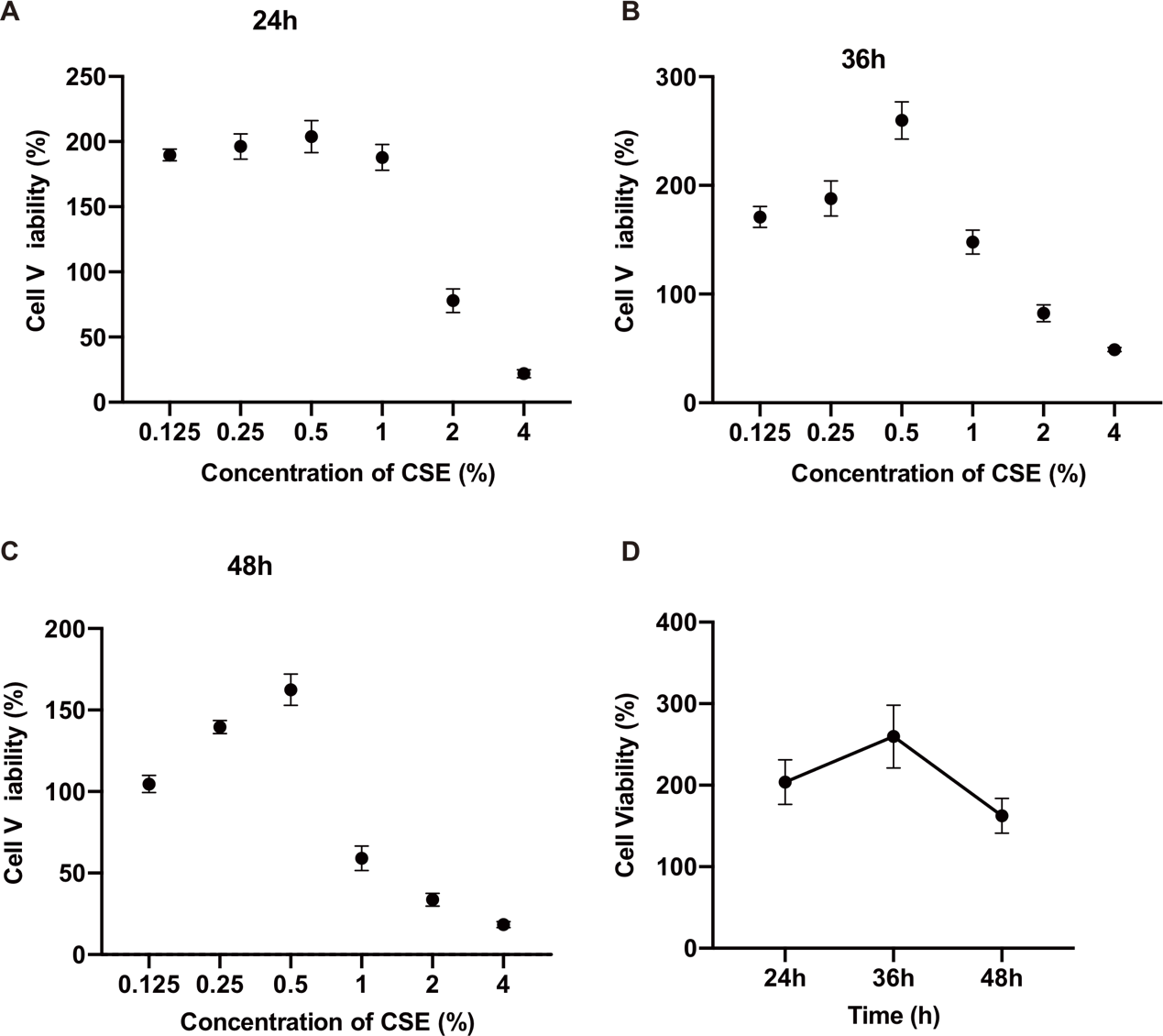
**

**Figure S3** Cell viability of human PASMCs stimulated by different CSE concentrations at different time points A：The cell viability of hPASMCs after 24 hrs stimulation with different CSE concentrations. B：The cell viability of hASMCs after 36 hrs stimulation with different concentrations of CSE. C：The cell viability of hPASMCs after 48 hrs stimulation with different concentrations of CSE. D: The cell viability of hPASMCs after stimulation with 0.5% at different time points. The abscissa of CSE represents the concentration (%) of CSE that stimulates human PASMCs. The ordinate represents cell activity (%); D: 0.5% concentration of CSE stimulates the cell activity of PASMCs at different time points. Abscissa, the time point of stimulating hPASMCs, ordinate, cell activity (%); N $=$5.
